# Alcohol-related liver disease and access to liver transplantation: where we stand and where we should go

**DOI:** 10.3389/ti.2026.16769

**Published:** 2026-08-27

**Authors:** Inês Mega, Eloisa Franchi, Giacomo Germani, Hermien Hartog, Speranta Iacob, Phillipe Mathurin, Silvio Nadalin, Stephen Potts, Giuliano Testa, Luca Saverio Belli

**Affiliations:** 1 Hepato-Biliary-Pancreatic and Transplantation Center, Curry Cabral Hospital, ULS São José, NOVA Medical School, Lisbon, Portugal; 2 General and Liver Transplant Surgery Unit, Foundation IRCCS Ca’ Granda Ospedale Maggiore Policlinico, Milan, Italy; 3 Department of Surgery, Oncology and Gastroenterology, Multivisceral Transplant Unit, Padua University Hospital, Padua, Italy; 4 Department of Surgery, Section of HPB and Liver Transplantation, University Medical Center Groningen, Groningen, Netherlands; 5 Carol Davila University of Medicine and Pharmacy, Center for Digestive Diseases and Liver Transplant, Fundeni Clinical Institute, Bucharest, Romania; 6 Center of Excellence in Translational Medicine, Fundeni Clinical Institute, Bucharest, Romania; 7 Inserm, CHU Lille, Université de Lille, U1286 INFINITE, Lille, France; 8 CHU Lille, Hôpital Claude Huriez, Service des Maladies de l’appareil Digestif, Lille, France; 9 Department of General, Visceral and Transplant Surgery, University Hospital Tübingen, Tübingen, Germany; 10 Transplant Psychiatry Department of Psychological Medicine, Royal Infirmary of Edinburgh, Edinburgh, United Kingdom; 11 Annette C. and Harold C. Simmons Transplant Institute Chief of Abdominal Transplant Baylor University Medical Center, Dallas, TX, United States; 12 Hepatology and Gastroenterology Unit, Azienda Socio Sanitaria Territoriale ‘Grande Ospedale Metropolitano’ Niguarda, Milan, Italy

**Keywords:** alcohol use disorder, alcohol-related liver disease, early transplantation, health equity, liver transplantation

## Abstract

Alcohol-related liver disease (ArLD) is now a leading indication for liver transplantation worldwide, yet access to this life-saving therapy remains inequitable. Late diagnosis, persistent stigma, rigid abstinence requirements, and fragmented care pathways continue to disadvantage patients with ArLD relative to those with other liver diseases. Landmark studies have shown that early liver transplantation for severe alcohol-associated hepatitis confers a clear survival benefit when performed within rigorous selection protocols, with rates of return to drinking comparable to those achieved under the long-standing six-month abstinence rule, thereby challenging its clinical rationale. Concurrently, growing evidence supports the integration of addiction medicine and mental health expertise into transplant programs, recognizing alcohol use disorder as a chronic relapsing disease requiring sustained, multidisciplinary management. Despite this progress, substantial variability persists across centers in candidate evaluation, psychosocial assessment, and post-transplant addiction care. This Point of View examines the current landscape across six interconnected domains, epidemiology, timing of diagnosis, the dual nature of ArLD as a disease of mind and liver, stigma, access to transplantation, and multidisciplinary models of care, and proposes directions for harmonized, evidence-based, and equitable practice.

## Highlights

- ArLD is now one of the leading indications for LT worldwide, with post-transplant outcomes comparable to those observed for other indications.
- ArLD is disproportionately diagnosed at late stages, reflecting missed opportunities in primary care and hospital settings where patients repeatedly present before decompensation.
- ArLD is a condition with a dual component, involving liver and mind; alcohol use disorder (AUD) is a chronic relapsing mental disorder requiring integrated psychiatric and addiction care alongside hepatological management.
- Stigma and perceptions of deservingness operate most powerfully upstream of allocation—at the stages of help-seeking, referral, evaluation, and listing—and disproportionately affect women, minorities, and socioeconomically disadvantaged patients.
- Early LT for severe alcohol-associated hepatitis confers a clear survival benefit within rigorous selection protocols, challenging the historical 6-month abstinence rule.
- Integration of addiction medicine into transplant programs and harmonization of evidence-based candidate evaluation are now essential for equitable access and improved long-term outcomes.

## Introduction

Liver transplantation (LT) is the most effective treatment for end-stage liver disease not amenable to medical therapy. Over the past 2 decades, the indications for LT have shifted profoundly, with alcohol-related liver disease (ArLD) emerging as one of the most frequent reasons for transplant listing and graft allocation in Europe and the United States [1–4]. This epidemiologic transition reflects the rising global burden of hazardous alcohol use and its hepatic consequences, but also an evolving willingness of the transplant community to reconsider longstanding eligibility barriers for patients with ArLD.

Despite generally favorable post-transplant outcomes comparable to those achieved for other indications [2, 4], patients with ArLD continue to face obstacles along the journey from diagnosis to listing, transplantation, and long-term follow-up. These obstacles are clinical, including late presentation, psychiatric comorbidity, and high short-term mortality in severe disease; structural, including geographic disparities, variable institutional policies, and fragmented addiction care; and societal, including stigma, moralised gatekeeping, and inequities related to gender, race, and socioeconomic status. Their cumulative effect is a landscape in which access to LT for ArLD is determined not by clinical need alone but by a complex interplay of medical, psychosocial, and systemic factors that too often disadvantage the most vulnerable patients.

This Point of View examines this landscape through six interconnected lenses, epidemiology, timing of diagnosis, the dual nature of ArLD as a disease of mind and liver, stigma, access to transplantation, and multidisciplinary models of care, drawing on recent registry data, prospective studies, consensus guidelines, and expert perspectives to synthesise where we currently stand and to propose concrete directions in where the field should go.

## Epidemiology: a shifting landscape

### Where we stand

ArLD is a leading cause of cirrhosis, hepatocellular carcinoma, and liver-related death, with a rapidly changing demographic profile. In the United States, ArLD mortality doubled from 1999 to 2022 (age-adjusted rates rising from 6.71 to 12.53 per 100,000), with the steepest acceleration during 2018–2022 and disproportionate increases among women, adults aged 25–44 years, and American Indian/Alaska Native populations [5]. Europe carries a similarly heavy burden: the WHO European Region reports approximately 800,000 alcohol-attributable deaths per year, with an estimated 50,000 deaths annually from cirrhosis and chronic liver disease attributable to high alcohol use, though substantial geographic heterogeneity is driven by consumption patterns and alcohol policy.^1^ A global epidemiological analysis spanning 2000–2021 confirmed the persistent and rising worldwide contribution of alcohol to liver morbidity and mortality [6].

This shift is striking among young adults and women: ArLD is now the leading LT-waitlisting indication among young American adults, overtaking all other etiologies [7], and adolescents and young adults increasingly present with alcohol-associated hepatitis [8]. The COVID-19 pandemic accelerated this trend, with rates continuing to rise even in its later phases [9].

These epidemiologic shifts have translated directly into rising demand for LT. Contemporary analyses describe ArLD as the most common indication for waitlist additions and transplantation in Europe [10] and the second most frequent in the United States since 2022, trailing only MASLD [11]. MetALD, combining metabolic dysfunction with moderate alcohol intake, is also emerging as a distinct, rapidly growing LT indication, with UNOS data showing an approximately three-fold rise in waitlisting and transplantation between 2002 and 2022, with worse post-transplant outcomes than ALD alone [12]. European registry analyses reveal substantial regional variation, with ArLD comprising a large share of transplants in some countries, reflecting differences in epidemiology, referral pathways, and center practices [10]. This growing demand occurs against a backdrop of persistent organ scarcity, amplifying the urgency for transparent and equitable allocation frameworks.

### Where we should go

Addressing this burden requires coordinated action at both population and healthcare-system levels. Strengthening public health policies on alcohol availability, pricing, and marketing remains the cornerstone of primary prevention. Within healthcare, integrating non-invasive fibrosis assessment and systematic screening for hazardous drinking into primary care and hospital admissions will enable earlier identification of patients at risk of advanced liver disease. Embedding the treatment of alcohol use disorder (AUD) into hepatology care pathways and building referral networks that connect early diagnosis with timely transplant evaluation are central health-system interventions with the potential to reduce both mortality and transplant need, supported by emerging integrated hepatology-addiction care models [13]. International collaboration will be essential to harmonize data collection and develop consensus strategies across transplant regions with differing alcohol burdens and healthcare infrastructures.

## Late diagnosis: too late, too often

### Where we stand

ArLD is still too often diagnosed after the windows for secondary and tertiary prevention have closed. Patients frequently present with decompensated cirrhosis or severe alcohol-associated hepatitis and acute-on-chronic liver failure, phenotypes associated with very high short-term mortality and a narrow opportunity for evaluation and intervention [3]. This late presentation appears to be a global pattern: in a large multinational cohort evaluating patients referred to specialist centers at different stages of liver disease progression, ArLD accounted for only 3.8% of early-stage presentations but for 29% of advanced-stage presentations; in the same study, patients with ArLD were approximately 14-fold more likely than those with hepatitis C to present with advanced-stage disease at specialist centers [14]. Real-world population linkage data from the United Kingdom paint a complementary picture: among 799 individuals dying from ArLD, more than 50% had no prior ArLD diagnosis or were diagnosed less than 6 months before death, even though the median patient had five hospital admissions and four emergency department attendances in the preceding five years—clear evidence of missed healthcare-system opportunities [15]. Delayed diagnosis also reflects patient-level self-stigma, which reduces help-seeking and disclosure of alcohol use [16], compounded by limited addiction-medicine training and inconsistent AUD screening among non-mental-health clinicians; stigma’s broader dimensions are discussed below.

Susceptibility to alcohol-related liver injury is heterogeneous. Only an estimated 10%–20% of heavy drinkers progress to cirrhosis, with genetic variants in PNPLA3, TM6SF2, and MBOAT7 increasing fibrosis risk, whereas loss-of-function HSD17B13 variants appear protective [3, 17]. Systematic prevalence synthesis confirms that while ArLD and cirrhosis are uncommon in general primary-care populations, they are far more frequent in cohorts with AUD, supporting targeted, risk-based case-finding rather than population-wide liver screening [18]. Conversely, alcohol-related liver damage may occur even without behavioral patterns meeting formal AUD criteria, highlighting that restricting evaluation to diagnosed AUD alone will miss patients with harmful or hazardous drinking who do not engage with addiction services [19].

### Where we should go

Addressing late diagnosis requires treating it as a public health problem and moving detection upstream, with dual screening for harmful alcohol use/AUD and fibrosis risk delivered where high-risk patients already present: primary care, hospital admissions, and addiction or mental health services, not only hepatology clinics. A 2024 systematic review confirmed that proactive case-finding works best in such high-risk settings, particularly acute hospitals and addiction services [20]. Population-level screening of at-risk groups offers the prospect of earlier intervention, delayed progression, and improved outcomes when individuals with advanced fibrosis or compensated cirrhosis are identified at a still-modifiable stage [21]. Current guidelines endorse a tiered, risk-based strategy: FIB-4 as a low-cost first-line triage in patients with metabolic risk or harmful alcohol use, followed by transient elastography (TE/FibroScan) in higher-risk individuals, with established stiffness measurement cut-offs to rule out (<8 kPa) and rule in (≥12–15 kPa) advanced fibrosis or cirrhosis [22]. Second-tier TE markedly improves specificity and reduces unnecessary referrals [23], and when deployed within addiction and community alcohol services it enables detection of clinically significant fibrosis at a pre-cirrhotic stage, as illustrated by a nurse-led outreach clinic in which 51% of attendees had abnormal liver stiffness and 38% had F2–F3 fibrosis [24].

**FIGURE 1:**
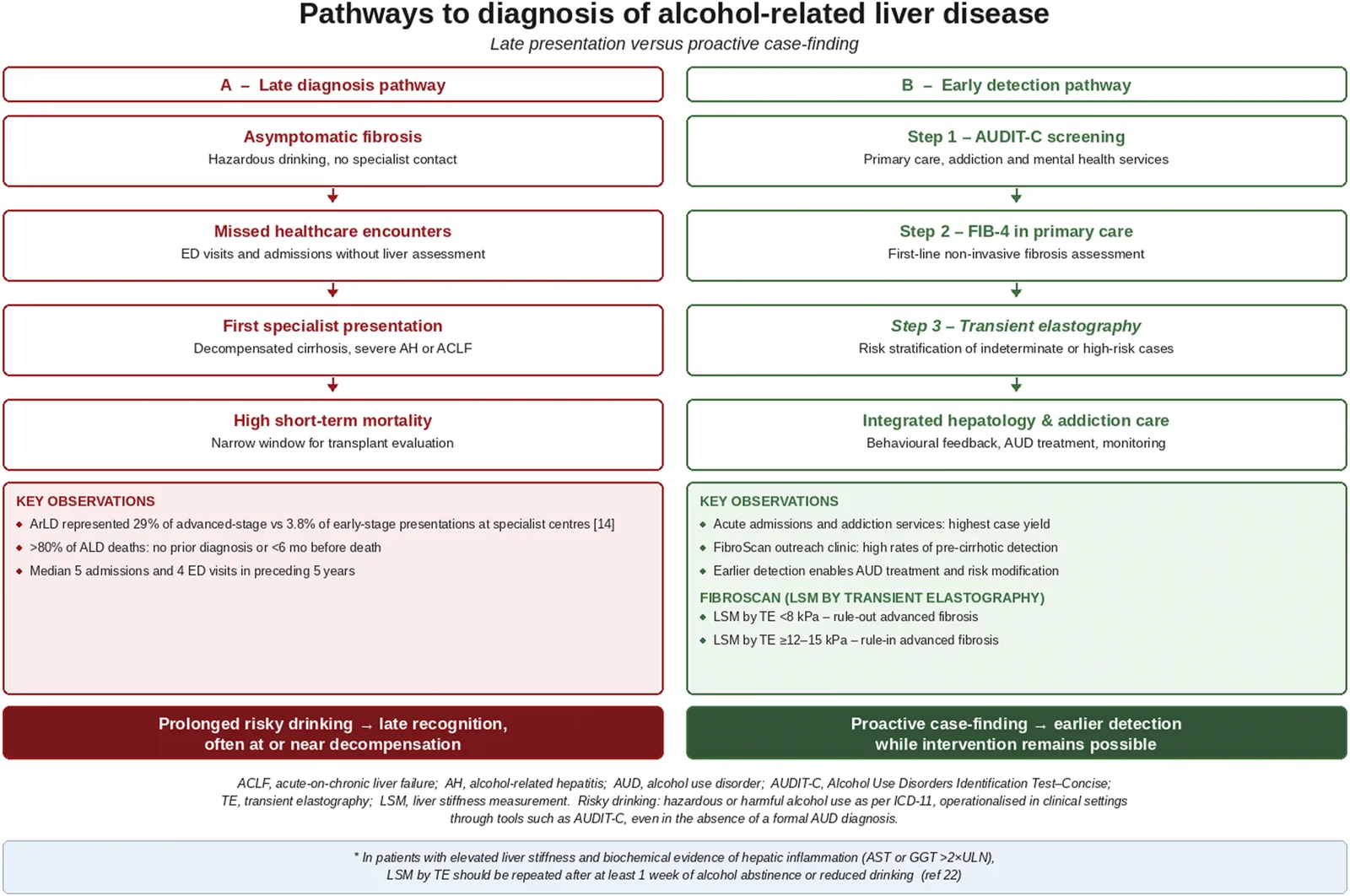
Pathways to diagnosis of alcohol-related liver disease: late presentation versus proactive case-finding. Panel A depicts the late-diagnosis pathway, in which prolonged unrecognized risky drinking leads to missed healthcare encounters and first specialist presentation only at the stage of decompensated cirrhosis, severe alcohol-associated hepatitis, or acute-on-chronic liver failure, with high short-term mortality and a narrow window for transplant evaluation. Panel B depicts a proactive case-finding pathway integrating AUDIT-C screening in primary care and addiction/mental health services, FIB-4 as first-line non-invasive fibrosis triage, transient elastography for risk stratification, and integrated hepatology-addiction care incorporating behavioral feedback, AUD treatment, and monitoring. Risky drinking denotes hazardous or harmful alcohol use as defined by ICD-11, operationalized in clinical practice through tools such as AUDIT-C, even in the absence of a formal AUD diagnosis. ACLF, acute-on-chronic liver failure; AH, alcohol-related hepatitis; AUD, alcohol use disorder; AUDIT-C, Alcohol Use Disorders Identification Test-Concise; TE, transient elastography; LSM, liver stiffness measurement.

Critically, early diagnosis alone is insufficient. It must trigger structured collaboration between hepatologists and mental health professionals to manage not only alcohol use but also coexisting conditions such as anxiety, depression, and personality factors that influence adherence, relapse risk, and liver outcomes. Risk stratification may further evolve to incorporate genetic susceptibility markers, enabling combined metabolic–genetic profiling to prioritise individuals at particularly high risk for closer monitoring [25]. Feedback of liver stiffness or fibrosis results to patients may support behavior change when paired with counseling and recovery-oriented support [26, 27] (Figure 1).

## A dual condition

### Where we stand

ArLD refers to a spectrum of alcohol-induced liver injury frequently comorbid with AUD. This comorbidity implies that patients have a condition caused by impairment on the liver and mind, and that clinicians need to treat both to be effective. AUD is present in the majority of patients with ArLD, with moderate-to-severe AUD affecting an estimated 75%–80% [28], and must not be considered a bad habit or moral failing, but a specific mental disorder requiring identification and treatment [29, 30]. AUD is substantially heritable (approximately 50% in twin and adoption studies) [31]; has well-identified neurobiological pathways involving reward and stress circuitry [32]; and is frequently precipitated by stressful life events including bereavement, separation, and unemployment [33].

A key and often underappreciated issue is that many patients with AUD harbour undiagnosed or untreated psychiatric conditions, depression, generalised anxiety disorder, panic disorder, post-traumatic stress disorder, that hepatologists are not well trained to diagnose and treat, and that may drive and sustain AUD [30, 34]. A frequent interplay exists between psychiatric illness, genetic predisposition, and stressful life events, with mental disorders and life events being potentially modifiable through medical treatment. Notably, undiagnosed depression is among the conditions most commonly leading to alcohol misuse, with alcohol serving as self-medication for profound suffering [29]. Training hepatology providers in AUD screening has been shown to improve both detection rates and resultant interventions [34].

### Where we should go

Integration of addiction care should begin upstream, in general hepatology clinics—the first point of contact for most patients with ArLD—where routine AUD screening and treatment access can help prevent progression to end-stage disease. Within the transplant setting specifically, centers should have a psychiatrist, psychologist, or addiction specialist embedded within their service to assess candidates and attend listing meetings. All patients with ArLD require a comprehensive psychosocial assessment of the nature and degree of underlying AUD, past and present, including psychiatric comorbidities, social support, and engagement with alcohol treatment services [35]. The primary purpose of assessment is to inform judgement about the risk of relapse to hazardous or harmful drinking after transplantation and to identify interventions to reduce that risk. For some patients, the risk may initially be so high as to contraindicate transplant listing; crucially, this does not mean it cannot be reduced, nor that such patients should be abandoned; an illustrative case is provided in Supplementary Material 1.

All clinicians in a liver transplant service need working familiarity with the main diagnostic criteria for AUD: DSM-5, which grades severity by symptom count (mild 2–3, moderate 4–5, severe 6 or more criteria in a 12-month period) [36], and ICD-11 [37], which classifies drinking into hazardous, harmful, and dependent patterns (Table 1). Assessment should identify which type of AUD (if any) the patient has, and the nature of any relationship to psychiatric comorbidities. This clinical framing then guides treatment decisions, including psychotropic medication, psychological therapies, pharmacological relapse prevention (e.g., acamprosate, naltrexone), and mutual support groups.

**TABLE 1:** Classification of alcohol use disorder according to the DSM-5 and ICD-11 diagnostic systems.

DSM-5: Alcohol use disorder	ICD-11: Disorders due to use of alcohol
Conceptual approach Single unified diagnosis (alcohol use disorder) replacing the former DSM-IV distinction between alcohol abuse and alcohol dependence. Severity is graded on a continuum by symptom count	Conceptual approach Retains categorical separation between distinct patterns of alcohol use: hazardous use (a health risk factor, not a disorder), harmful pattern of use, and alcohol dependence. Each category has distinct diagnostic requirements
Diagnostic criteria Two or more of the following in a 12-month period: • Alcohol taken in larger amounts or over longer periods than intended • Persistent desire or unsuccessful efforts to cut down or control use • Excessive time spent obtaining, using, or recovering from alcohol • Craving or strong desire/urge to use alcohol • Recurrent use resulting in failure to fulfil major role obligations • Continued use despite persistent social or interpersonal problems • Important social, occupational, or recreational activities given up or reduced • Recurrent use in situations where it is physically hazardous • Continued use despite knowledge of a persistent physical or psychological problem likely caused or exacerbated by alcohol • Tolerance (need for increased amounts or diminished effect) • Withdrawal symptoms or use of alcohol to relieve/avoid withdrawal	Hazardous use of alcohol A pattern of alcohol use that appreciably increases the risk of harmful consequences to the user, without current harm. Listed as a health risk factor (QE11.2), not a mental disorder Harmful pattern of use of alcohol A pattern of alcohol use that has caused clinically significant harm to physical or mental health or has resulted in behavior leading to harm to the health of others. May be episodic or continuous Alcohol dependence Requires two or more of the following three core features: • Impaired control over alcohol use (onset, level, circumstances, or termination) • Increasing priority given to alcohol use over other activities, such that use becomes a dominant activity in daily life • Physiological features indicative of neuroadaptation: Tolerance to the effects of alcohol, or withdrawal symptoms upon cessation or reduction Features usually evident over ≥12 months, or ≥3 months if use is continuous (daily or almost daily). May be episodic, continuous, or in remission
Severity grading • Mild: 2–3 criteria • Moderate: 4–5 criteria • Severe: 6 or more criteria	Severity grading Severity is implicit in the categorical distinction: • Hazardous use: risk without current harm • Harmful pattern of use: documented harm • Dependence: compulsive use with neuroadaptation
Key differences relevant to transplant assessment • DSM-5 uses a single continuum of severity, facilitating standardized grading of AUD across the full spectrum of disease • ICD-11 maintains categorical separation, distinguishing between harmful use (with or without dependence) and dependence itself, which may be more informative for risk stratification in transplant candidates • By definition, all patients with ArLD have drinking patterns that are at least harmful, but not all meet criteria for dependence—a distinction with direct implications for treatment planning and relapse risk assessment	

AUD, alcohol use disorder; ArLD, alcohol-related liver disease; DSM-5, Diagnostic and Statistical Manual of Mental Disorders, 5th edition; ICD-11, International Classification of Diseases, 11th revision.

## Stigma, deservingness, and the gatekeeping of care

### Where we stand

ArLD is a paradigmatic setting in which moral narratives can become clinical thresholds for referral, evaluation, listing and prioritization. Stigma in this context is not simply individual clinician bias; it is a social process that crystallises when labeling, stereotyping, separation, and resulting status loss converge within a power situation that enables unequal outcomes [38]. In ArLD, the label is culturally saturated and readily tethered to stereotypes, weak-willed, unreliable, shaping differential treatment long before formal allocation rules apply.

The adjacent concept of deservingness captures how assumptions about whose health merits attention and care shape healthcare provision and can render some groups less deserving despite substantial need [39]. In transplantation, deservingness becomes clinically active because the resource is necessarily scarce: the supply of donor livers is finite, demand exceeds supply, and selection is unavoidable. A key attribute shifting perceived deservingness is whether liver failure is seen as self-inflicted. Even when teams reject overt moralism, deservingness can return indirectly through two expectation sets: engagement (“they will not show up, they will not adhere to immunosuppression”) and outcome (“they will drink the graft away”). These beliefs can distort thresholds, intensify demands for proof of insight or change, and justify delayed referral, an under-recognized form of discrimination that may occur before transplant centers are even involved [16, 40].

Critically, stigma operates most powerfully not in post-listing allocation, designed to be as objective as possible, but upstream: at the stages of help-seeking, referral, evaluation, and listing. It is intersectional and amplified where discretion is widest, as psychosocial risk assessment can expand subjective judgement, particularly at lower MELD scores. Women, especially Hispanic and Asian women, with ArLD are less frequently listed for LT compared with men [41, 42], while racial and ethnic minorities in North America experience lower listing rates and higher wait-list mortality, reflecting structural inequalities in healthcare access and broader social determinants of health [43–45]. The historical 6-month abstinence rule, initially linked to potential hepatic recovery rather than prediction of future drinking, can function as a barrier for patients too sick to survive the waiting period, a further illustration of how structural rules may encode moralised gatekeeping.

### Where we should go

The field must move toward making stigma measurable rather than mystical. First, a stronger evidence base is needed to demonstrate that for diagnoses carrying moral stigma, thorough assessment and protocol-based candidate selection can yield outcomes comparable to those for non-stigmatised conditions, not merely as a technical exercise, but as an antidote to the common sense of stigma. Second, opportunity-cost arguments must be examined rigorously: time-pressured assessments may grant the benefit of the doubt to some groups while demanding extended proof of change from others, an asymmetry that may look like fairness but operate as bias. Third, transplant systems should audit referral delays, listing rates, psychosocial assessment thresholds, and post-transplant support allocation across aetiologies, while explicitly addressing the persistent stigma attached to obesity and to comorbidities such as alcohol use with metabolic-associated steatotic liver disease. If stigma thrives at the boundaries of discretion, then transparency, protocolisation, and equity-focused outcomes tracking are not administrative add-ons—they are core transplant ethics [6, 16, 46].

## Access to liver transplantation: from the 6-month rule to early transplantation

### Where we stand

For many years, transplant eligibility for ArLD was governed by the 6-month abstinence rule, which required documented abstinence before listing. This policy was widely adopted to allow potential hepatic recovery after alcohol cessation and to provide a pragmatic measure of commitment to sobriety [47, 48]. However, the scientific basis for this rule has always been limited, and abstinence duration alone is an imperfect predictor of post-transplant relapse, which is instead influenced by a broader range of psychosocial and behavioral factors [49–51].

A major paradigm shift occurred with the landmark study in 2011, demonstrating that early LT could provide a substantial survival benefit in carefully selected patients with severe alcohol-associated hepatitis (AH) who failed medical therapy (6-month survival: 77% vs. 23% in matched controls; p < 0.001) [52]. Subsequent studies confirmed these findings: single-center and multicenter experiences in Europe and North America reported one-year survival rates of 89%–100%, comparable to outcomes observed for other transplant indications [53–56]. The American multicenter study involving 147 patients across 12 centers, reported cumulative patient survival of 94% at 1 year and 84% at 3 years [53]. A multicenter Italian study of 93 patients demonstrated 100% survival at 6, 12, 24, and 36 months, with survival significantly higher in transplanted patients than in those denied LT [55].

The prospective Franco-Belgian controlled study provided the first head-to-head comparison of drinking behavior after early versus standard LT, using a standardized psychosocial assessment algorithm. The proportion of patients returning to any alcohol use was 34% after early LT compared with 25% after standard transplantation; sustained harmful drinking, however, remained relatively uncommon when candidates were selected through rigorous evaluation [57]. These data underscore the nuanced nature of post-transplant drinking and the inadequacy of binary distinctions between abstinence and relapse, while highlighting strict candidates selection as a central key for favorable outcomes.

Despite these advances, access to LT for ArLD remains heterogeneous across programs and geographic regions. Some centers have abandoned fixed abstinence requirements, whereas others continue to apply minimum abstinence periods, reflecting differences in institutional culture, perceptions of relapse risk, and ethical perspectives regarding organ allocation [47, 48, 58]. This shift has progressed unevenly: US practice has moved decisively away from mandatory abstinence, though local variability persists [59]; European centers, similarly no longer mandating fixed abstinence [47], show comparably wide heterogeneity. Geographic disparities, socioeconomic disadvantage, limited health literacy, and restricted access to specialised hepatology care further compound these inequities [60–62]. At a population level, nationwide data after first decompensation demonstrate lower transplant probability and higher mortality for alcohol-related cirrhosis, with female sex and social deprivation independently associated with reduced access [63]. Psychosocial evaluation has been shown to exert greater influence than severity of liver failure on selection for LT listing among ArLD candidates [64], highlighting how non-biological factors can gatekeep access even in advanced disease.

### Where we should go

Looking forward, transplant programs should replace rigid abstinence-based criteria with harmonized, evidence-based candidate evaluation frameworks built around structured psychosocial assessment and relapse prediction tools such as the Sustained Alcohol Use Post-Liver Transplant (SALT) score, a four-item risk instrument [49]; external validation, however, shows only moderate accuracy and limited generalisability, so SALT should inform, rather than replace, comprehensive assessment [65]. These assessments should inform, not exclude: psychiatric comorbidity *per se* should not preclude LT, and should instead guide targeted intervention and individualised listing decisions in the context of liver disease severity and transplant urgency. Reducing disparities in access must become a parallel priority, addressing socioeconomic barriers, geographic variability, and the racial and ethnic inequities that shape listing and waitlist mortality [61–64]. International collaboration is needed to establish consensus recommendations and facilitate equitable access across regions [57]. Further research should refine relapse prediction models, clarify long-term outcomes after early LT for severe AH, and evaluate interventions that support recovery from AUD after transplantation [44–46]. Ultimately, the future of LT for ArLD will depend on balancing responsible stewardship of scarce donor organs with equitable access to life-saving therapy [60]. The integration of addiction medicine into transplant care, essential to this vision, is addressed in the next section.

## The multidisciplinary model of care

### Where we stand

Despite widespread recognition of AUD as a chronic relapsing disease affecting mind and behavior, the integration of mental health and addiction expertise into LT programs for ArLD remains inconsistent across institutions. Psychosocial evaluation is universally acknowledged as a cornerstone of transplant candidacy assessment, often viewed as the most difficult and contentious part of a comprehensive evaluation [35], and although a multidisciplinary model of care is increasingly seen as essential, its composition, depth, and process vary markedly between centers [66]. Decision-making regarding eligibility, often binary, is frequently influenced by subjective judgements, bias, or rigid abstinence requirements rather than structured, validated tools and evidence-based clinical rationale [67].

Surveys reveal that fewer than 50% of transplant centers have defined protocols for managing return to alcohol use after transplantation, and fewer than 40% have explicit metrics to evaluate outcomes in this population [68]. This lack of standardization undermines equity in access, disproportionately affecting women, minority groups, and patients with comorbid psychiatric disorders, and perpetuates stigma-driven decision-making [41]. The transplant literature lacks a uniform approach to AUD diagnosis and risk stratification, largely driven by the uneven distribution of addiction expertise and institutional resources [69]. Most centers continue to outsource post-transplant addiction care to community services, fragmenting the continuum of care at one of the most critical junctures in a patient’s recovery [68].

### Where we should go

The evidence increasingly supports the integration of dedicated psychiatrists, psychologists, social workers, and addiction specialists as core members of the LT multidisciplinary team, working alongside hepatologists and surgeons throughout the entire transplant process [35, 70, 71]. These professionals should be equipped to assess AUD severity, psychiatric comorbidities, motivation for sobriety, and relapse risk using validated instruments, such as the Stanford Integrated Psychosocial Assessment for Transplant (SIPAT), the High-Risk Alcoholism Relapse (HRAR) scale, or the SALT score, transforming listing decisions from intuitive to evidence-based [35, 66]. Co-located, collaborative models in which stakeholders jointly discuss each candidate have demonstrated feasibility and improved outcomes, including reduced admissions and improved MELD-Na scores [72]. One of the first dedicated multidisciplinary pathways combining addiction treatment with transplant evaluation reported low relapse rates [73]; eligibility should be understood as dynamic rather than fixed, since declined candidates can later be re-evaluated once risk factors are addressed [74].

Beyond listing decisions, the multidisciplinary team must extend its role into the post-transplant setting. Relapse prevention therapy forms the core psychotherapeutic approach to AUD after transplantation, whereas motivational interviewing retains its principal role pre-transplant and may be revisited should post-transplant alcohol use recur. Pharmacotherapy (naltrexone, acamprosate, baclofen, gabapentin) and peer support programs should be offered systematically, as integrated multimodal care reduces return to alcohol use and improves graft outcomes [69, 75–77]. Advocating for this model is not merely a clinical imperative but an ethical one, ensuring every patient with ArLD receives fair evaluation and the sustained support needed for lasting recovery.

## Conclusion

The approach to LT for ArLD has undergone profound transformation over the past decade. The historical reliance on fixed abstinence periods is increasingly replaced by individualised psychosocial evaluation and multidisciplinary management of AUD. Yet this transformation remains incomplete and unevenly distributed. Late diagnosis, persistent stigma, fragmented addiction care, and structural inequities continue to shape a landscape in which access to transplantation is determined by factors that extend well beyond clinical severity.

The path forward requires coordinated action across several fronts: upstream screening and early diagnosis linked to integrated addiction and mental healthcare; transparent, protocol-based psychosocial evaluation that minimizes the influence of bias and stigma; harmonized candidate selection criteria replacing rigid abstinence rules with evidence-based risk stratification; addiction medicine embedded as a core component of transplant programs before and after transplantation; and systematic efforts to measure and mitigate disparities related to sex, race, socioeconomic status, and geography. Only by addressing the full continuum of care, from primary prevention to long-term post-transplant recovery, can the transplant community ensure that patients with ArLD receive the equitable, evidence-based care they deserve.

## Data Availability

The original contributions presented in the study are included in the article/Supplementary Material, further inquiries can be directed to the corresponding author.

## Footnotes

1 World Health Organization. Alcohol use: data and statistics (2024). Available from: https://www.who.int/europe/news-room/fact-sheets/item/alcohol-use [Accessed March 2026].
