# Supplementary material for: Alcohol-related liver disease and access to liver transplantation: where we stand and where we should go
Source: Transpl Int. 2026 Aug 27;39:16769. doi: 10.3389/ti.2026.16769 (PMC13558123; doi:10.3389/ti.2026.16769)
Supplement: Supplementary file 1 [file Supplementaryfile1.docx]

# Supplementary Material

**Supplementary File 1. Clinical vignette illustrating the role of embedded psychiatric care in liver transplant evaluation.**

A 56-year-old man with alcohol-related liver disease (ArLD) secondary to alcohol use disorder (AUD) achieved abstinence through his own efforts after a diagnosis of cirrhosis. He had no contact with alcohol services and limited social support. The transplant psychiatrist identified a recurrent depressive illness with prominent anxiety, for which no treatment had been initiated. She informed the listing meeting that the relapse risk was too high to proceed with listing and initiated antidepressant therapy, referral to community mental health services, and a relapse-prevention program with acamprosate. With these interventions, the risk of relapse was judged to have fallen sufficiently to justify listing for liver transplantation.

*This vignette illustrates how structured psychiatric assessment within a multidisciplinary transplant team can identify modifiable psychiatric comorbidity, reduce relapse risk, and enable equitable access to liver transplantation for patients initially judged unsuitable.*
